# Reconstruction of the cropland cover changes in eastern China between the 10^th^ century and 13^th^ century using historical documents

**DOI:** 10.1038/s41598-018-31807-6

**Published:** 2018-09-10

**Authors:** Meijiao Li, Fanneng He, Shicheng Li, Fan Yang

**Affiliations:** 1 Key Laboratory of Land Surface Pattern and Simulation, Institute of Geographic Sciences and Natural Resources Research, Chinese Academy of Sciences, Beijing, China; 2 University of Chinese Academy of Sciences, Beijing, China; 3 School of Public Administration, China University of Geosciences, Wuhan, China

## Abstract

To evaluate and improve datasets of anthropogenic land cover change used in local and global climate models, great efforts were made to reconstruct historical land use, including the LandCover 6k project which dedicated to reconstructing human land use over the past 10,000 years. In this study, we utilized historical records, including taxed-cropland and cropland measurement areas, and data on the number of households in eastern China between the 10^th^ century and 13^th^ century in concert with coefficient calibration, model allocation, and per capita cropland estimation to reconstruct areas of provincial cropland for 22 provinces over five time periods. Our reconstructions indicate that total cropland areas of eastern China for AD 1000, 1066, 1078, 1162, and 1215 are 34.74 × 10^6^ ha, 49.42 × 10^6^ ha, 51.62 × 10^6^ ha, 35.21 × 10^6^ ha, and 51.21 × 10^6^ ha, respectively. And the cropland area fluctuated because of dynasty shift and went through three phases. Cropland expansion and contraction mainly occurred in the middle and lower reaches of the Yangtze and Yellow Rivers as well as the Huaihe River Basin, while in some regions far away from battlefields, including northeastern and southern China, cropland area expanded continuously throughout the study period.

## Introduction

Historical anthropogenic land use and land cover change (LULCC) has led to significant modifications to the natural environment^1–5^ and has influenced climate change via biogeochemical and biogeophysical mechanisms^6–11^. At present, human activities are estimated to have transformed or degraded approximately 50% of the land surface, mostly through agriculture^12^. Therefore, among all the types of land use, agricultural activity plays an important role in controlling land surface transformation and greenhouse gases emissions. For example, the transformation of non-cropland to cropland could alter the land-atmosphere exchange of energy and water and thereby change temperature, humidity, precipitation, convection and wind speed^13,14^. Moreover, the emission of greenhouse gases by the agriculture ecosystem account for approximately 50% and 60% of the total global CH_4_ and N_2_O anthropogenic emissions, respectively^15^. Thus, datasets recording patterns of historical cropland are essential to modeling global environmental change and understanding the impact of land use conversion on the temporal dynamics of environmental and ecological issues^16–19^.

A number of notable research achievements in understanding historical LULCC have been attained in recent years in the context of global research projects such as the Land-Use and Land-Cover Change (LUCC) project, the Global Land Project (GLP), Past Global Changes (PAGES), LandCover 6k, and Future Earth. Three representative global scale datasets encompassing the last millennium have been established, including the Historical Database of the Global Environment (HYDE), which was produced by the Netherlands Environmental Assessment Agency^20,21^. This dataset has been updated several times, and the latest version (HYDE3.2) encompasses the period between 10,000 BC and AD 2015. Using the land cover maps for AD 1700 from the SAGE dataset^22^ and historical population data as a proxy, Pongratz *et al*.^23^ reconstructed cropland and pastureland cover for the period between AD 800 and AD 1700 (the PJ dataset), while Kaplan *et al*.^24,25^ reconstructed an anthropogenic LULCC dataset (KK10) that covers the period between 8000 BP and AD 1850. However, because the global land use areas estimated by these global datasets are based on historical population records and the relationships between population and land use, there are large differences and uncertainties in the total areas and spatial distributions of global land use from one dataset to another^20,25^. For instance, Klein Goldewijk *et al*.^20^ reported that the PJ dataset estimated ~10% more total global cropland area than the HYDE dataset for the period of AD 800–1700. In addition, Kaplan *et al*.^25^ reported that the HYDE dataset estimate of the global land areas under anthropogenic land cover change was ~80% lower than that of the KK10 dataset for the preindustrial era. Moreover, some researchers have also illustrated that these datasets have many uncertainties at the regional scale and do not reflect the trends and characteristics of land use change in China^26–29^ and Europe^30^. As a result, in order to better understand regional LULCC and to simulate the resultant effects on climate and ecological change, a number of researchers worldwide have attempted to reconstruct regional areas of historical cropland using available local historical documents^31–34^.

China boasts an ancient civilization, a long history, and abundant historical documents. The presence of these historical materials provides a sound basis for the study of historical cropland. Thus, across the country, and especially in eastern China, significant progress reconstructing historical cropland areas at national and regional scales based on local historical documents has been made by a number of researchers in recent years. For example, the provincial cropland areas of China over the last 300 years and in the middle period of the Northern Song Dynasty (AD 1078) were reconstructed by utilizing historical documents, modern surveys, and inventory data^35–38^. At the same time, a number of high-resolution reconstructions also exist at the regional scale, including estimates for provincial and county-level cropland areas in northeastern China^39^, Shandong Province^40^, Zhili Province^41^, and Zhenlai County^42^ for the last 300 years. In addition, based on local chronicles, Zhao^43^ and Fu^44^ estimated the county-level cropland areas in Huizhou and Henan Province between AD 1500 and AD 1900 and AD 1368 and AD 1953, respectively.

The bulk of the research noted above encompasses the last three centuries, and just a handful of studies have covered the Ming (between AD 1368 and AD 1644) and Qing (between AD 1644 and AD 1912) Dynasties. Thus far, a historical document-based reconstruction of provincial-level land cover that encompasses the whole of eastern China between the 10^th^ century and the 13^th^ century has not been carried for a number of reasons. First, this region was ruled by multiple nationalities and regimes throughout the 10^th^ and 13^th^ centuries. This means that a diverse range of historical documents are available, as the regimes and territories frequently changed, rendering LULCC research for this period complex. In addition, the available historical documents related to land use from the Liao (between AD 907 and AD 1125), Song (between AD 960 and AD 1279), and Jin Dynasties (between AD 1115 and AD 1234) are not as rich as those from the Ming and Qing Dynasties. It has thus proven hard to reconstruct patterns in LULCC for China between the 10^th^ century and 13^th^ century. However, reconstructing the anthropogenic land use and land cover for these periods is essential to understand the relationship between humans and atmospheric CO_2_ in the preindustrial era, because, in the first few centuries of the last millennium, Asia experienced large-scale anthropogenic land cover change, and it has been hypothesized that the rapid increase in atmospheric CO_2_ concentrations that occurred at that time were related to anthropogenic deforestation^45^.

The aim of this study is to use historical documents to reconstruct the cropland area of eastern China between the 10^th^ century and 13^th^ century at the provincial level. Thus, by analyzing land and tax systems, population policies, the history of administrative settings, the characteristics of taxed-cropland area, the crop yield, and the human demand for grain, we established a series of cropland area estimation methods using traditional historical documents for the Liao (between AD 907 and AD 1125), Song (between AD 960 and AD 1279), and Jin Dynasties (between AD 1115 and AD 1234). We then used these results to reconstruct the provincial cropland area of eastern China between the 10^th^ century and 13^th^ century. The results of this study are significant to reconstructions of historical LULCC over the last millennium and provide basic data enabling simulations of the effects of regional climatic and ecological factors on historical LULCC over this time period.

## Study Area

**Figure 1:**
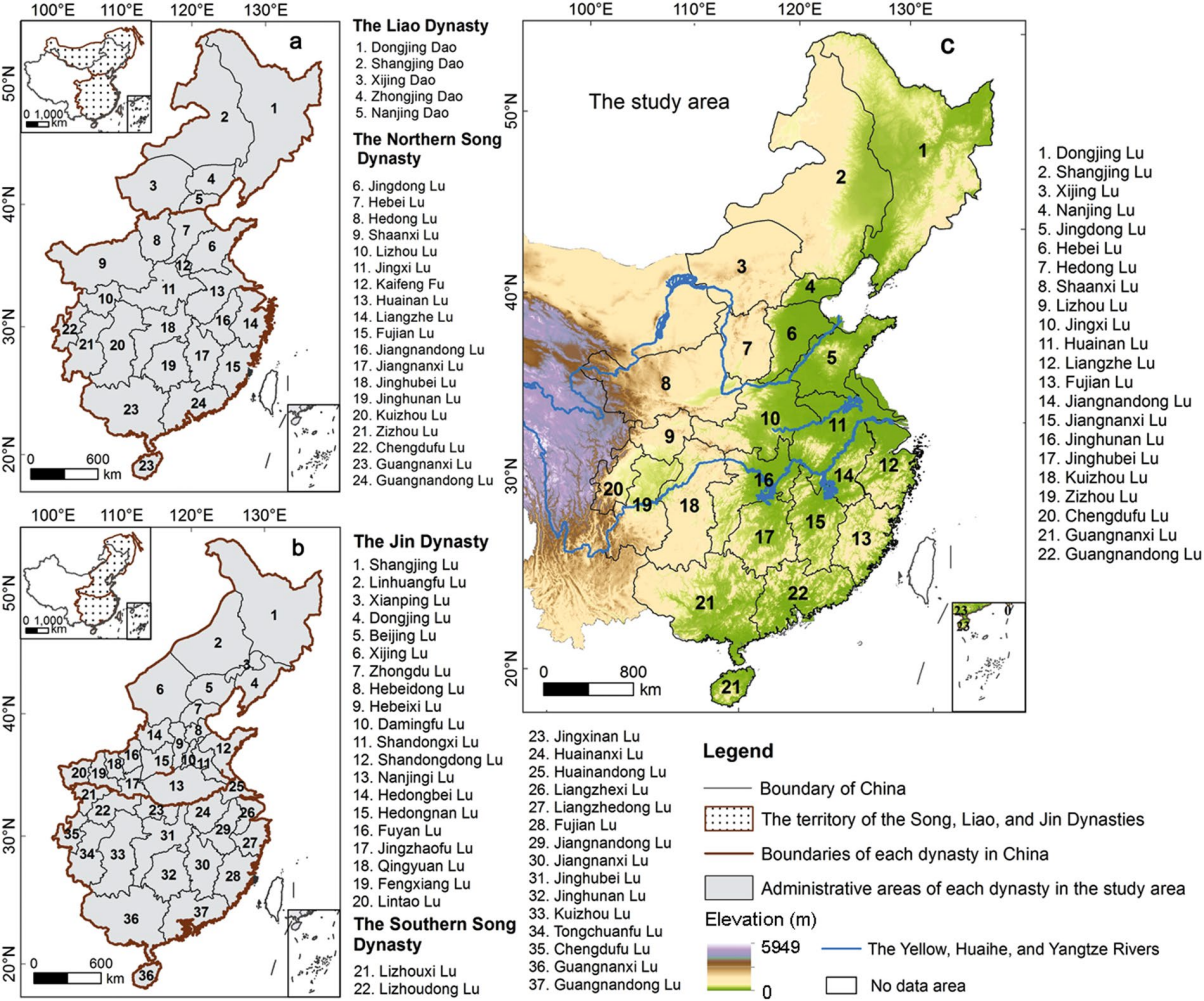
Maps of eastern China. (**a**) Time period encompassed by the Northern Song and Liao Dynasties. (**b**) Time period encompassed by the Southern Song and Jin Dynasties. (**c**) Summary map of our study area. From the north to south, the blue lines denote the locations of the Yellow, Huaihe, and Yangtze Rivers in present-day China.

This study focuses on eastern China (Fig. 1), a region between the latitudes 3°51′N and 53°33′N and the longitudes 100°28′E and 135°05′E, which covers an area of approximately 4.49 × 10^8^ ha. The main environments within this region are plains and mountains, and the region demonstrates a great deal of environmental variability; extensive and densely populated alluvial plains characterize the east, while the western areas are dominated by hills and mountain ranges. Eastern China has a humid and semi humid monsoonal climate, and the major climatic zones are temperate and subtropical. As the average annual precipitation ranges between 400 mm and 2,000 mm, the natural conditions are very conducive to agricultural development; thus, eastern China is viewed as a traditionally cultivated region of the country.

It is well-known that from the end of the 10^th^ century to the early 13^th^ century, eastern mainland China was ruled by the Liao (between AD 907 and AD 1125), Song (between AD 960 and AD 1279), and Jin (between AD 1115 and AD 1234) Dynasties (Fig. 1a,b) and that external and internal boundaries changed frequently in the period of these three regimes. Thus, we use the contemporary national boundaries of China in this study and denote the provincial administrative boundaries of the Northern Song and Liao Dynasties as our geographic framework, adjusting these boundaries accordingly in the other two historic periods. To reduce uncertainties in the reconstruction results, we also adjusted and merged contiguous provincial administrative areas, selecting a ‘Lu’ (a provincial-level political unit in use during the Song and Jin Dynasties) as a basal area unit for analysis. A total of 22 Lu*-*level units were therefore employed in this study (Fig. 1c).

## Results

### Overall changes in cropland area between the 10^th^ century and 13^th^ century

**Figure 2:**
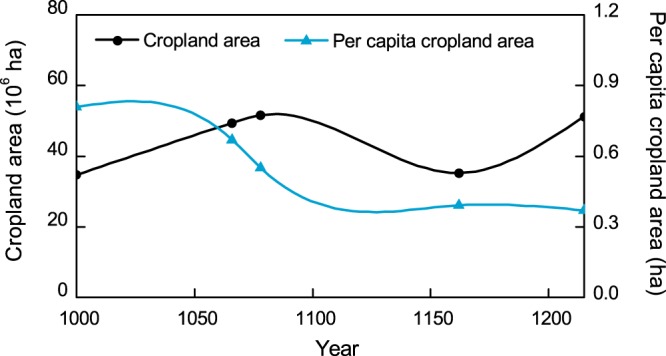
Plots showing the changes in cropland area and per capita cropland area across eastern China between the 10^th^ century and the 13^th^ century.

Our reconstructions indicate that total cropland areas of eastern China for AD 1000, 1066, 1078, 1162, and 1215 are 34.74 × 10^6^ ha, 49.42 × 10^6^ ha, 51.62 × 10^6^ ha, 35.21 × 10^6^ ha, and 51.21 × 10^6^ ha, respectively. And the per capita cropland areas are 0.81 ha, 0.67 ha, 0.55 ha, 0.39 ha, and 0.37 ha, respectively. The results of this study show that trends in the cropland area across eastern China fluctuated between the 10^th^ century and the 13^th^ century and can be characterized by three phases of development, while the per capita cropland area decreased continuously over the study period (Fig. 2).

The first of these phases of development is a rapid increase in cropland area between the end of the 10^th^ century and the late 11^th^ century; during this period, the total cropland area increased from 34.74 × 10^6^ ha (AD 1000) to 51.62 × 10^6^ ha (AD 1078), the maximum value estimated for our study period. At the same time, the fractional cropland area (FCA) increased from 7.7% to 11.5%, reflecting an average annual growth rate of 0.51%. Although eastern China at this time was dominated by the Northern Song and Liao Dynasties, the war between the two ended following the establishment of the *Chanyuan Alliance* () armistice in AD 1004. Society remained relatively stable in both dynasties in the aftermath of war, and the population of eastern China gradually increased from 43.0 million to 93.7 million, while agriculture also recovered and developed.

Over the next 80 years, between AD 1078 and AD 1162, eastern China experienced a regime change and a period of upheaval. Throughout this period, the regimes in eastern China transitioned from the Northern Song and Liao Dynasties to the Southern Song and Jin Dynasties, and wars between the three dynasties continued for decades from the early 12^th^ century onwards. As a result, large numbers of people migrated from the north to the south; the population south of the Yangtze River increased from 49.7 million to 59.4 million, and cropland destruction of the north became extremely serious. The results show that the total cropland area of eastern China sharply decreased to 35.21 × 10^6^ ha at this time (AD 1162), a reduction of approximately 16.41 × 10^6^ ha over 80 years. At the same time, the FCA decreased from 11.5% (AD 1078) to 7.8% (AD 1162), reflecting an annual average growth rate of −0.45%.

The final phase in eastern Chinese cropland development started in the middle of the 12^th^ century (AD 1162) when the available cropland area increased to 51.21 × 10^6^ ha (AD 1215), an increase of approximately 16.01 × 10^6^ ha over 50 years. The FCA also recovered to 11.4% over this period (AD 1215), reflecting an annual average growth rate of 0.71%. The *Longxing Peace Treaty* () between the Jin and Southern Song Dynasties was signed in AD 1163 and marked the end of conflicts and the start of a period of peace in eastern China. Society gradually stabilized, the population increased from 91.1 million to 139.1 million, and the government enacted a series of policies to promote immigration, land cultivation, and the growth of land returns.

### Provincial-level changes in cropland area

**Figure 3:**
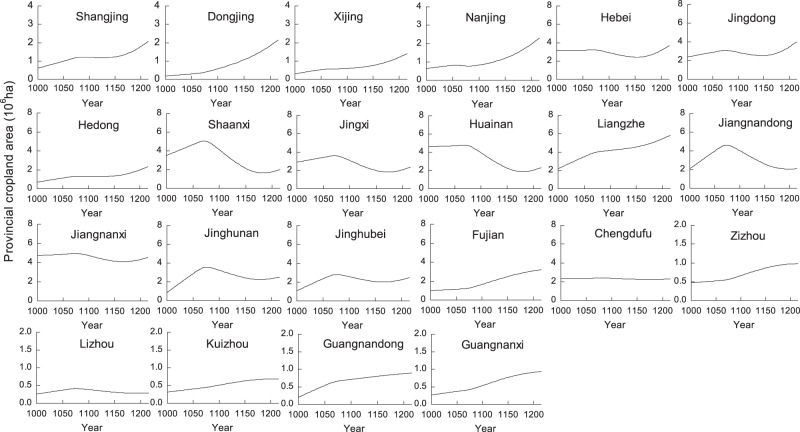
Graphs showing the reconstructed provincial cropland areas for the period between the 10^th^ century and the 13^th^ century.

The results show that the changes in cropland area at the provincial level vary among Lus (Fig. 3). Data show that variations in cropland area in the middle and lower reaches of the Yellow River (including the Hebei, Jingdong, Hedong, Shaanxi, and Nanjing Lus), in the Huaihe River Basin (including the Jingxi, Huainan, Lizhou, and Jinghubei Lus), and in the middle and lower reaches of the Yangtze River (including the Jiangnandong, Jiangnanxi, and Jinghunan Lus) conform to almost the same trends as those seen across the whole of eastern China, including a rapid increase between AD 1000 and AD 1078, a rapid decrease between AD 1078 and AD 1162, and an additional sharp increase between AD 1162 and AD 1215.

However, in northeastern China (including the Shangjing, Dongjing, and Xijing Lus), in the Liangzhe and Fujian Lus, and in the southwestern part of the country (including the Zizhou, Kuizhou, Guangnandong, and Guangnanxi Lus), the trends in cropland area change deviate from those in eastern China, as cropland area increased continuously over the study period. Especially after AD 1078, following the migrations of people out of war areas, the available cropland area in these regions increased markedly. Indeed, the average annual growth rates in the northeastern and southwestern regions were 0.72% and 0.48%, respectively, and after AD 1078, the annual growth rate in the northeast peaked at 0.76%. In the Liangzhe and Fujian Lus, the annual cropland area growth rates were 0.45% and 0.55%, respectively, while this rate in the Fujian Lu reached 0.69% after AD 1078.

### Changes in the spatial distribution of cropland cover

**Figure 4:**
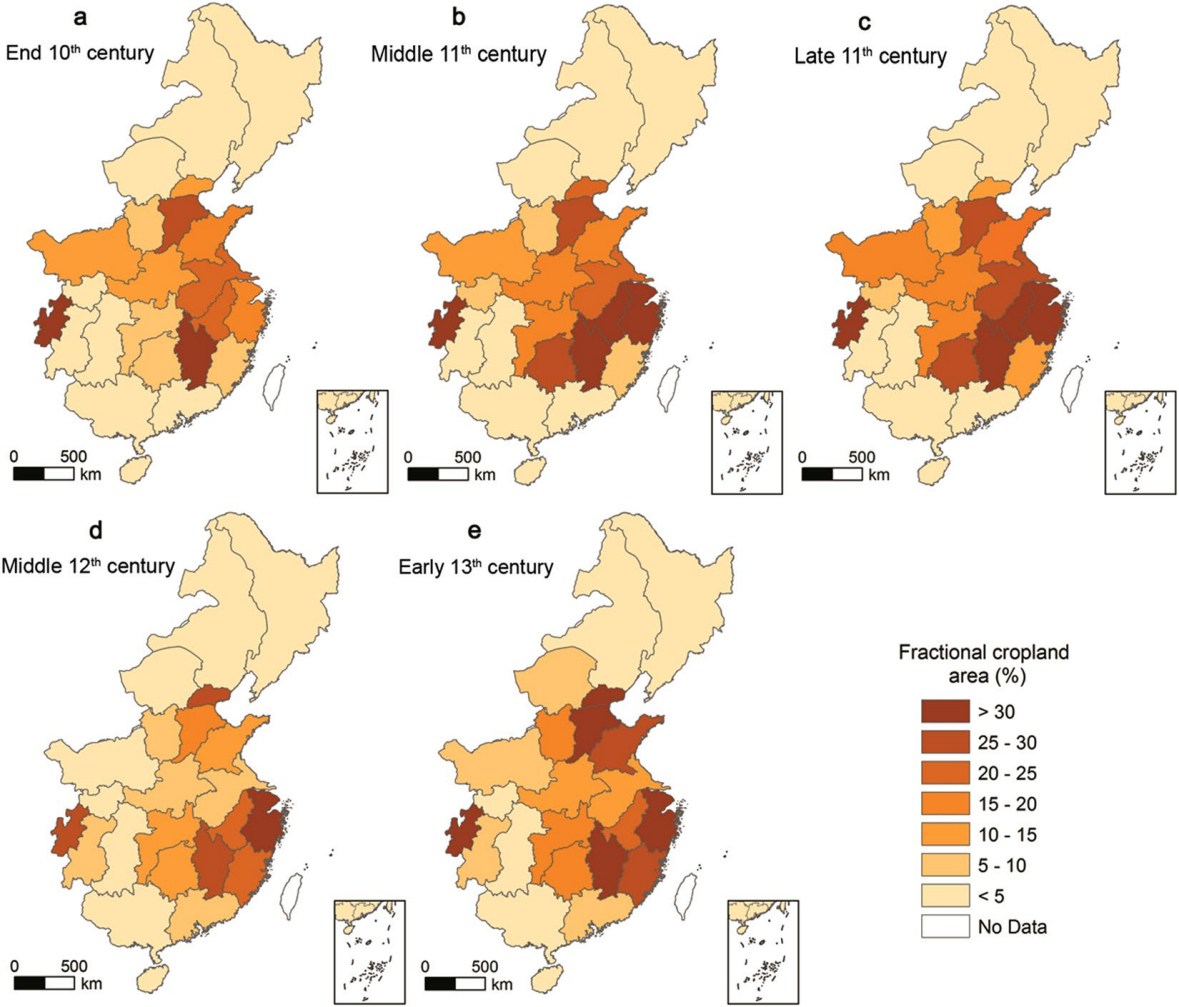
Maps showing the reconstructed FCA results from the 10^th^ to 13^th^ centuries.

**Figure 5:**
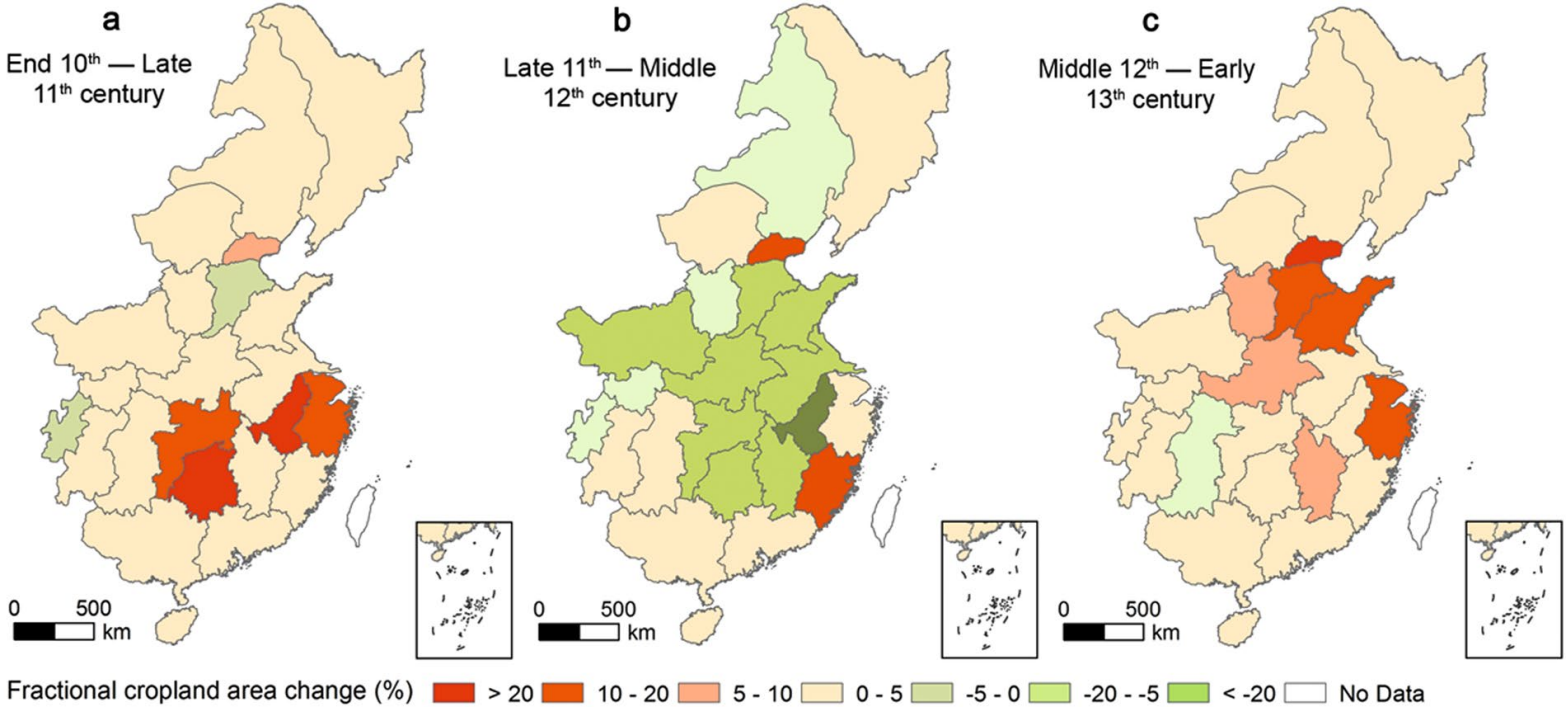
Maps showing the changes in the FCAs of each Lu in eastern China from the 10^th^ to 13^th^ centuries.

To better understand the spatial characteristics of cropland changes at the provincial level, we calculated the spatial distribution of FCA in each of the 22 Lus across eastern China by dividing the cropland area by the total land area. We then calculated changes in FCA values for the periods between AD 1000 and AD 1078, AD 1078 and AD 1162, and AD 1162 and AD 1215 (Figs 4 and 5).

Data show that traditional agricultural areas with high FCA values include the middle and lower reaches of the Yellow and Yangtze Rivers, the Huaihe River and the Chengdu plains. The FCA values for most Lus in these areas were greater than 15% (Fig. 4), while they were less than 5% for most Lus in the northeastern and southwestern regions. The spatial distribution of FCAs between AD 1000 and AD 1078 is consistent with the earlier pattern (AD 1000) (Fig. 4c), while the FCA values for the middle and lower reaches of the Yangtze River increased markedly over this period of peace in concert with population growth. The FCAs of the Jiangnandong, Liangzhe, and Jinghunan Lus increased by approximately 31%, 15%, and 22% (Fig. 5a), respectively, over this period, and increased to more than 35% by AD 1078. At the same time, in the middle and lower reaches of the Yellow River, FCA values increased by less than 5% because of ongoing wars between the Northern Song and Liao Dynasties (Fig. 5a). In AD 1078, however, the FCAs of many Lus increased to more than 20% in concert with intensified farming activities and population movements outwards from the North China plain to the Yangtze River plains (Fig. 4c). Data show that the FCAs of the northeastern and southwestern regions also expended gradually with the migration of the agricultural population; however, the FCAs for most Lus in these regions remained less than 5% because of unfavorable terrain and climatic conditions.

The FCAs of traditional agricultural areas decreased markedly between AD 1078 and AD1162 as a result of wars between the Jin, Liao, and Song Dynasties, especially the continuous conflicts between the Jin and Song regimes, which culminated in the well-known *Jingkang Shame*. At the frontier of the war between the Southern Song and Jin Dynasties, the FCA values notably decreased by more than 10% at this time, declining from greater than 21% to less than 9% (Fig. 5b) in the main agricultural region comprising the Huaihe River Basin and its surrounding areas. Similarly, in the middle and lower reaches of the Yellow and Yangtze Rivers, the FCAs of most Lus also decreased; however, in some areas far removed from the battlefield (including in Liangzhe, Fujian, Guangnandong, Guangnanxi, Zizhou, and Kuizhou in the south and Dongjing, Xijing, and Nanjing in the north), the FCAs continued to increase because of population movements away from the war zone. The results show that by AD 1162, the FCAs of the Liangzhe and Fujian Lus had increased to more than 30% and 25%, respectively, while the FCA values in the southwest had increased to more than 5% (Fig. 4d).

After AD 1160, because of the influence of the *Longxing Peace Treaty*, a new environment led to a recovery and expansion in croplands, and the FCAs of a large number of Lus gradually increased (Fig. 5c). This trend was especially marked in the middle and lower reaches of the Yangtze and Yellow Rivers, as the agricultural policies implemented by the Jin and Southern Song Dynasties led to rapid land reclamation. Inside Jin Dynasty territories, the FCAs of the Hebei and Shandong Lus increased by more than 10% (Fig. 5c), while in the Nanjing Lu, the capital of thisdynasty, FCAs increased by more than 20%. The FCA values for the Nanjing Lu and its surrounding areas had increased to more than 20% by AD 1215 (Fig. 4e). Similarly, land reclamation was also marked along the middle and lower reaches of the Yangtze River plain, the central region of the Southern Song Dynasty. The results show that the FCA in Liangzhe and Jiangxi increased by more than 5% and 10%, respectively, at this time, while the extent of land reclamation in the northeastern and southwestern regions of China also expanded, and the values for most Lus increased to more than 5%. However, because of the severe impact of the *Jingkang Shame*, FCA values in the Huaihe River Basin remained lower than 15%, which was less than the FCA value at the end of the 11^th^ century (Fig. 4c–e).

## Discussion

To date, most studies reconstructing historical LULCC in China have addressed the LULCC in just the last three centuries. This study is the first to reconstruct the provincial cropland area across eastern China over the 10^th^ to 13^th^ centuries. One notable aspect of this research is our use of abundant Chinese historical records to study LULCC.

A number of global LULCC datasets, including the PJ^23^, HYDE^20,21^, and KK10^25^ datasets, encompass the whole of eastern China and our study time period. Because the KK10 dataset estimated global land areas under anthropogenic land cover change instead of cropland areas, we only compared our results with the cropland areas from the PJ and HYDE datasets. In terms of methods, the PJ dataset reconstructed cropland areas by making the assumption that the ratio of area utilized per capita for crops did not change in each country prior to AD 1700; thus, this dataset uses an AD 1700 per capita cropland area of approximately 0.14 ha for China. However, as highlighted by the HYDE dataset, per capita values for cropland are not constant; rather, they may slightly increase or decrease over time.This means that results based on the PJ dataset could be underestimated. Based on this conclusion, the per capita cropland values over the past 12,000 years were first estimated country-by-country by the HYDE dataset before national- and provincial-level cropland areas were reconstructed using historical population estimates and per capita cropland values^20,21^. However, as the HYDE dataset is global in scale, its time resolution is too coarse for a local-scale study; indeed, the per capita cropland areas for China in our study time period reported by HYDE remained constant, with a value of approximately 0.15 ha.

We comprehensively analyzed abundant historical documents, taxed-cropland area data, household numbers and population data, regime shifts, tax systems, and changes in land policies over our chosen time period to reconstruct the characteristics of taxed-cropland, as well as the relationships between cropland area and the number of households or the population at a regional scale for each dynasty. We then reconstructed provincial cropland areas for five time periods in eastern China. Our data reveal clear differences in the per capita cropland area at the regional and provincial level for each dynasty over the study period. Overall, our data show that the per capita cropland area of eastern China decreased from 0.81 ha to 0.37 ha (Fig. 2), and a significantly different result than those recovered by the PJ and HYDE datasets. Notably, the lowest per capita value for cropland area in our reconstruction was 1.6 and 1.4 times higher than those reported by the PJ and HYDE datasets, respectively. This is an important difference because this variable is the main parameter used in reconstructions of historical cropland area; any variation in per capita cropland area will lead to significant differences in the results of reconstructions.

For instance, using the per capita cropland areas of the PJ and HYDE datasets and Chinese population estimates (Supplementary S1.2), we calculated the total cropland areas derived from the PJ and HYDE datasets for eastern China and compared the values with our results. For the PJ and HYDE datasets, the total cropland area increased from 6.20 × 10^6^ ha (AD 1000) to 18.14 × 10^6^ ha (AD 1200) and 6.45 × 10^6^ ha to 19.44 × 10^6^ ha, respectively. In contrast, in this study, the total cropland area increased from 34.74 × 10^6^ ha to 51.21 × 10^6^ ha. Comparison results show that the relative differences between this study and two global datasets are greater than 60%. As discussed by Kaplan^45^, these uncertainties may influence the calculation result of CO_2_ emission in eastern China in the study period.

Our comparative results also show that reconstructing a regional LULCC dataset on the basis of historical facts from different areas leads to more reliable data. Adopting a historical approach not only significantly advances our understanding of environmental effects but also contributes to enriching and improving the precision of global LULCC datasets. As discussed by Ramankutty and Coomes^46^, the reconstruction of regional land use areas that result from regime shifts has the potential to significantly advance our understanding of the LULCC dynamics that drive global change.

However, several uncertainties still exist in this study. There are many uncertainties associated with the calibration coefficient estimates between the real cropland and the registered taxed-cropland areas for the Northern Song Dynasty. For this period, historical records on taxed-cropland areas are abundant, and data on cropland measurement areas are reliable; therefore, we calibrated the national and provincial taxed-cropland areas based on the ratios between the sporadically registered taxed-cropland areas and the cropland measurement areas. However, the value of the calibration coefficient for the south was only determined using sporadic data in the Liangzhe Lu, based on the assumption that this value was approximately the same in each Lu in the south. However, calibration coefficient values may vary by Lu and over time. Therefore, additional historical records on the cropland measurement area during the Song Dynasty will help to improve the quality of cropland reconstructions by further clarifying the ratio between the real cropland area and registered taxed-cropland area.

A similar statement can be made for the per capita cropland area estimates. Although the per capita cropland areas were estimated in this study by utilizing historical records and the estimated results were very different for each region, while the per capita cropland area estimates were constant in eastern China for the global dataset, the accuracy of the per capita cropland area estimates can still be improved. For the Liao, Jin, and Southern Song Dynasties, there are only historical data on household numbers and population and sporadic historical records on the taxed-cropland areas, so estimating the cropland areas using the per capita cropland area is practical. Therefore, the per capita value of cropland areas is an important, sensitive parameter in total cropland area reconstructions for eastern China over the 12^th^ to 13^th^ centuries. In this paper, we estimated the per capita cropland area based on historical records, depending on different regions. And the results show that the per capita cropland area estimates varied from 0.27 ha to 1.13 ha across different regions during the 12^th^ century to 13^th^ century. However, in the estimation of the per capita cropland area of frontier areas, including the southwest and northeast of China, we assumed that the per capita grain demand maintained a basic life level and that the crop yield remained constant from the 10^th^ century to the 13^th^ century. And this estimation method might have resulted in the underestimation of the frontier cropland areas. Consequently, collecting more historical records on taxed-cropland areas, household numbers, per capita grain demand, and crop yield will be helpful for improving the accuracy of per capita cropland area estimates and cropland area reconstructions.

In addition, the population estimates of some Lus in the frontier areas and some time periods with missing data are questionable. For eastern China, there are reliable historical data on population and household numbers in *The History of Chinese Population*^47^. Chinese scholars calibrated and estimated provincial household and population data based on historical records. However, for some Lus in frontier areas with administrative boundaries beyond the current Chinese national boundaries, we estimated the population by using the ratio between the inner area and the whole area, based on the assumption that the population was evenly distributed throughout. This approach may have underestimated the population and cropland areas in the frontier areas because there is a close relationship between the distribution of people and the natural environment. Moreover, the number of households and the population during some time periods for some regions with missing data were calculated in this study using interpolation methods. Both of these procedures for processing data may lead to variations in reconstructions.

## Methods

**Figure 6:**
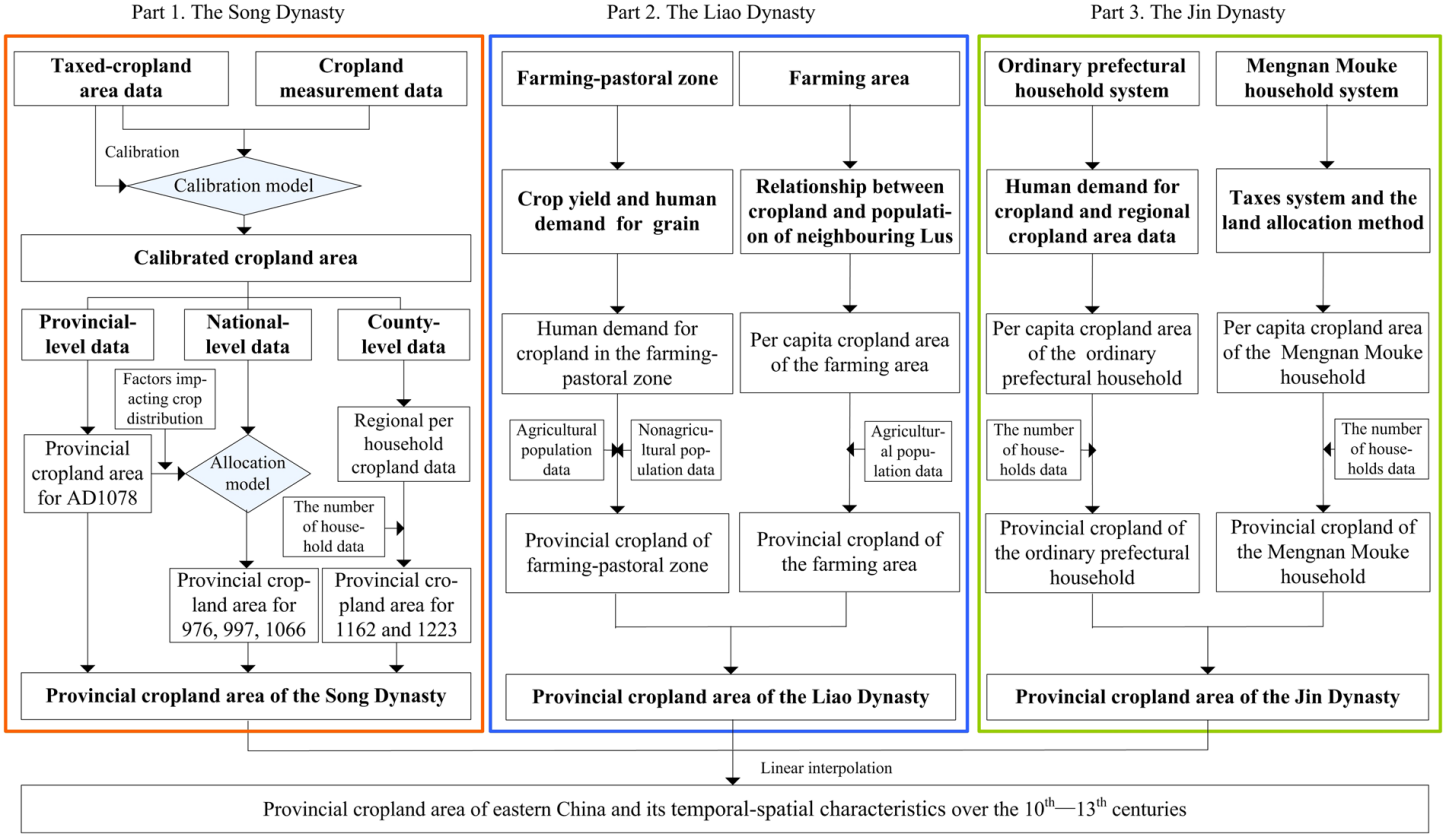
Overview of inputs leading to provincial cropland area reconstructions for the period between the end of the 10^th^ century and the early 13^th^ century.

The Song (including the Northern and Southern Song), Liao, and Jin Dynasties were ruled by the Han (), Khitan (), and Jurchen () nationalities, respectively. As a result, there were significant differences in the population compositions and the policies related to land-use and taxation systems among the three regimes, while the taxation basis, corvée levied, historical records related to cropland area, the number of households and population also vary markedly. We therefore developed a series of methods to calibrate and estimate provincial cropland cover during the time periods of the three dynasties (Fig. 6).

### Reconstructing the provincial cropland area of the Song Dynasty

Using the provincial taxed-cropland area data for AD 1078, the national taxed-cropland area data for AD 976, 997, and 1066, the sporadic county-level taxed-cropland area data for the Southern Song Dynasty (AD 1127–1279), the sporadic cropland measurement data for AD 1085 and AD 1189 (Supplementary S1.1), and household and population data estimates (Supplementary S1.2), the provincial cropland areas of the Song Dynasty were reconstructed. There were three major steps in the reconstruction:

(1) Calibrating the provincial taxed-cropland area in AD 1078. Using the provincial taxed-cropland area for AD 1078 and the above described sporadic cropland measurement data, we calculated the ratios between the real cropland areas and the taxed-cropland areas in the north and south during the Song Dynasty. Then, these ratios were used as calibration coefficients to convert the registered taxed-cropland area to the real cropland area, as follows:

1 $${C}_{kr}=\alpha \cdot ({C}_{r}/{C}_{t})\cdot {C}_{kt}$$

where *α* is the ratio between unit areas of mu (a Chinese unit of area, where1 mu = 0.067 ha) and Song-mu (an area unit used by the Northern Song Dynasty, where 1 Song-mu = 0.058 ha) and has a value of 0.876^48^. The ratio between *C*_*r*_ and *C*_*t*_ is 2.03 for the north and 1.93 for the south^38^.

**Figure 7:**
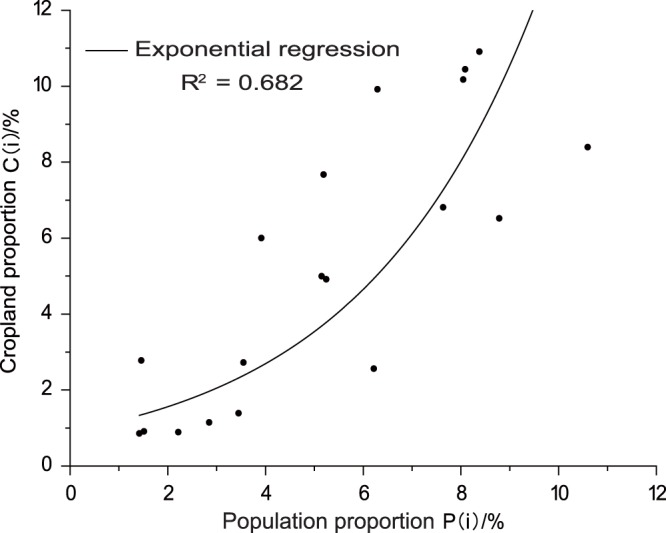
The curve-fitting relationship between cropland and population proportions in the middle of the Northern Song Dynasty period.

(2) Reconstructing provincial cropland areas in AD 976, AD 997, and AD 1066. We subsequently used our cropland calibration ratio for AD 1078 to calibrate the national cropland areas for AD 976, AD 997, and AD 1066, with Lu-level impact factors selected for the cropland distributions (i.e., altitude, slope, and population distribution), and analyzed their relationships. The results of this analysis show that population was the most important factor influencing cropland distribution at the Lu level; thus, a higher population and per capita cropland resource value in one Lu translates to a higher probability of cropland distribution (Fig. 7). We therefore designed a Lu-level cropland area allocation model based on Equation (2), as follows:

2 $$X(i,{t}_{v})=\frac{{C}_{p}(i)/{\rm{\max }}({C}_{p}(i))\times 0.904\times \exp (0.273\cdot P(i,{t}_{v}))}{{\sum }_{i}{C}_{p}(i)/{\rm{\max }}({C}_{p}(i))\times 0.904\times \exp (0.273\cdot P(i,{t}_{v}))}\times A({t}_{v})$$

where *X*(*i*, *t*_*v*_) denotes the cropland area of Lu *i* in year *t*_*v*_, *P*(*i*, *t*_*v*_) indicates the population proportion of *Lu i* in year *t*_*v*_, *A*(*t*_*v*_) denotes the total cropland area in year *t*_*v*_, *C*_*p*_(*i*) denotes the per capita cropland area of Lu *i* in AD 1078, and max(*C*_*p*_(*i*)) denotes the maximum value of *C*_*p*_(*i*).

As discussed, we used the calibrated national cropland area for AD 1078 allocated at the provincial level to initially validate our model. We then compared our allocated results with the calibrated provincial cropland area for AD 1078; the comparisons indicate that Lus with differences less than 10% account for as much as 68.40% of the total, while 15.80% exhibit differences larger than 20%. These results suggest that our model provides an appropriate transformation from national-level to provincial-level cropland areas. This approach was therefore used to transform the national-level cropland area estimates for AD 976, AD 997, and AD 1066 to the provincial level.

**Figure 8:**
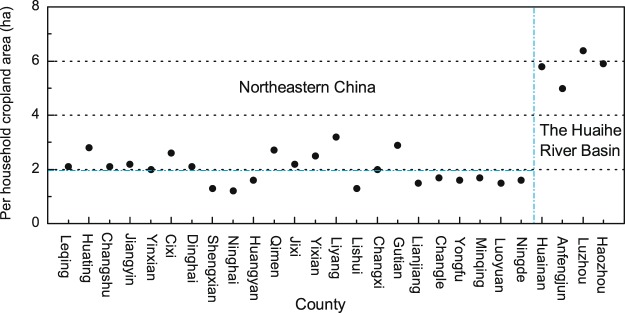
County-level cropland areas per household in southeastern China and the Huaihe River Basin for the Southern Song Dynasty; the horizontal blue dotted line denotes the average value of cropland area per household in southeastern China; the vertical blue dotted line separates southeastern China from the Huaihe River Basin.

(3) Estimating provincial cropland areas in AD 1162 and AD 1223. Based on the data characteristics for this period, we estimated the cropland areas for AD 1162 and AD 1223 using the cropland areas per household. We initially calibrated the sporadic county-level taxed-cropland data points using calibration coefficients from the south. Then, the proportional relationship between cropland area and the number of households was analyzed using these county-level data. The results show that cropland areas per household can be approximated in the southeast and in the Huaihe River Basin (Fig. 8). Therefore, we reconstructed the provincial cropland areas of these two regions using household data for AD 1162 and AD 1223 and produced values of 2.02 ha in southeastern China and 5.87 ha in the Huaihe River Basin. We also estimated the missing values for the southwest using household data and the human demand for cropland, applying a cropland estimation method that we have described previously^38^.

### Reconstructing provincial cropland area during the Liao Dynasty

Regarding the Liao Dynasty, there are only population estimates for the mid to late Liao Dynasty (AD 1078), and there are no known historical records on cropland areas. Given this lack of primary data, we estimated the cropland area for this period on the basis of an empirical relationship between population and cropland using typical factors for crop yield and human grain demand (Supplementary S3.3). For farming-pastoral zones (i.e., Shangjing, Zhongjing, and Dongjing Daos), there are significant differences in the human grain demand between agricultural and nonagricultural populations, so the population compositions were first analyzed in this region (Supplementary S3.2). For farming areas (i.e., Nanjing and Xijing Daos), the provincial cropland areas were estimated using agricultural population data and the per capita cropland area of the Hedong and Hebei Lus during the middle period of the Northern Song Dynasty (AD 1078), based on the argument that the relationship between cropland and population of one region should be similar to that of another region with a comparable geographic environment. We estimated the total provincial cropland area in AD 1078 using Equations (3) and (4), as follows:

3 $${C}_{k}=\alpha \times {P}_{ka}\times (P{G}_{ky}/{G}_{ky})+\alpha \times {P}_{kn}\times (P{G}_{ky}/{G}_{ky})\times 1/10$$

4 $${C}_{k}=\alpha \times {P}_{ka}\times P{C}_{i}$$

where *C*_*k*_ denotes the total cropland area of Dao *k*, *P*_*ka*_ indicates the agricultural population of Dao *k*, *PG*_*ky*_ denotes the human grain demand in one year, *G*_*ky*_ is the crop yield of Dao *k*, *P*_*kn*_ is the nonagricultural population of *Dao k*, and *PC*_*i*_ refers to the per capita cropland area of the Hedong and Hebei Lus during the middle period of the Northern Song Dynasty (AD 1078).

### Reconstructing provincial cropland area during the Jin Dynasty

For the Jin Dynasty, only provincial data on the number of households in AD 1207 and sporadic data about cropland areas in North China were recorded in historical documents. Reconstructing the provincial cropland area for this period using per capita cropland area is a pragmatic approach. The population of the Jin Dynasty was consistently composed of Han and Jurchen nationalities, so the government created two local administrative organizations to manage the two groups, an ordinary prefectural household and Mengan Mouke. Two different taxation and land systems were also implemented to collect revenue and manage lands. These differences resulted in a large gap in the per capita value of cropland areas between Mengan Mouke and ordinary prefectural households. Therefore, the population compositions for this period were first analyzed in this paper (Supplementary S4.1).

Subsequently, the per capita cropland areas of two types of households were calculated. These values were estimated by using the local crop yield and human grain demand of the northeast (Supplementary S3.3) and the regional cropland data of North China (Supplementary S4.3) for ordinary prefectural households, while for Mengnan Mouke () (a Jin Dynasty form of local administrative organization), this value was determined based on their tax system and cropland allocation method (Supplementary S4.2). Finally, we estimated the provincial cropland area for AD 1207 of the Jin Dynasty using Equation (5), as follows:

5 $${C}_{k}=\alpha \times ({H}_{k}-{H}_{m})\times {\beta }_{p}\times P{C}_{p}+\alpha \times {H}_{m}\times {\beta }_{m}\times P{C}_{m}$$

where *C*_*k*_ denotes the total cropland area of Lu *k*, *H*_*k*_ and *H*_*m*_ represent the number of ordinary prefectural and Mengan Mouke households in Lu *k*, respectively, and *β* is the conversion coefficient. *β* values of 6.5 and 10 were used for the ordinary prefectural (*β*_*p*_) and Mengan Mouke households (*β*_*m*_), respectively. *PC*_*p*_ denotes the per capita cropland area (or the human cropland demand) of each ordinary prefectural household in northern China, and *PC*_*m*_ represents the per capita cropland area of each Mengan Mouke household.

### Harmonizing reconstruction periods

We have reconstructed provincial cropland areas in AD 976, AD 997, AD 1066, and AD 1078 during the Northern Song Dynasty, in AD 1162 and AD 1223 during the Southern Song Dynasty, in AD 1078 during the Liao Dynasty, and in AD 1207 during the Jin Dynasty. These results show that although the territories of the Northern Song and Liao dynasties coexisted in space within eastern China, their time periods of reconstructed cropland are mismatched. As a similar situation is also present when the Southern Song and Jin dynasties are compared, we performed linear interpolations based on population growth rates^47^ to harmonize the results using the boundaries presented in Fig. 1c and developed a series of 22 provincial cropland area reconstructions encompassing the end of the 10^th^ century (AD 1000), the middle and late of the 11^th^ century (AD 1066, AD 1078), the middle of the 12^th^ century (AD 1162), and the early 13^th^ century (AD 1215).

## Electronic supplementary material

Supplementary information

## Data Availability

The datasets generated during and/or analyzed during the current study are available from the corresponding author on reasonable request.
