# Supplementary material for: Reconstruction of the cropland cover changes in eastern China between the 10th century and 13th century using historical documents
Source: Sci Rep. 2018 Sep 10;8:13552. doi: 10.1038/s41598-018-31807-6 (PMC6131514; doi:10.1038/s41598-018-31807-6)
Supplement: Supplementary file 1 — Supplementary information [file 41598_2018_31807_MOESM1_ESM.pdf]

Supplementary information

Reconstruction of the cropland cover changes in eastern China between the 10th century and 13th century using historical documents

Meijiao Li [1, 2], Fanneng He [1*], Shicheng Li [3], Fan Yang [1, 2]

[1] Key Laboratory of Land Surface Pattern and Simulation, Institute of Geographic Sciences and Natural Resources Research, Chinese Academy of Sciences. Beijing, China

[2] University of Chinese Academy of Sciences, Beijing, China

[3] School of Public Administration, China University of Geosciences, Wuhan, China

Contents

S1 Data sources **2**

S1.1 Collection of cropland area data 2

S1.2 Household and population data 3

S2 The Song Dynasty **3**

S2.1 Accuracy assessment of cropland area data 3

S3 The Liao Dynasty **4**

S3.1 Taxation systems 4

S3.2 Population compositions 5

S3.3 Human demands for cropland 5

S4 The Jin Dynasty **6**

S4.1 Population compositions 7

S4.2 Cropland allocation method for Mengan Mouke households 7

S4.3 Per capita cropland area for ordinary prefectural households 8

S1 Data sources

S1.1 Collection of cropland area data

These data include taxed-cropland area and cropland measurement data. It is noteworthy that while the taxed-cropland area recorded in historical documents do not correspond to actual areas [1,2] but an indicator of agricultural taxes. However, as these values nevertheless bear close relationship with real cropland areas, they are the raw materials that form the basis for our estimates. In contrast, cropland measurement areas are real as these were recorded by officials appointed by the government to increase national taxes. These measurements, however, were only carried out in limited areas, and so data are sporadic. We therefore utilized several available measurements for cropland areas in this study in order to estimate the relationship between taxed-cropland and real cropland areas as accurately as possible.

National and provincial level taxed-cropland area data were mainly obtained from the *Collections of Historical Governmental Archives* (《文献通考》). This volume contains provincial taxed-cropland area data (19 Lu) for AD 1078 as well as national taxed-cropland area data for AD 976, AD 997, AD 1066, and AD 1078 and thus encompasses the period between the end of the10th century and the early 13th century. County level taxed-cropland area data was derived mainly from *Collections of Local Chronicles of the Song and Yuan Dynasties* as well as the *Collected Texts of the Administrative Statues of the Song Dynasty* (《宋会要辑稿》). The former volume comprises approximately 29 categories of local Song Dynasty chronicles; we derived taxed-cropland area data for 23 southeastern counties from nine local chronicles from the Southern Song Dynasty.

In addition, cropland measurement data for five northern Lus, including Kaifeng, Jingdong, Hebei, Hedong, and Shaanxi, were derived from the *Monograph on Food and Property from the History of the Song Dynasty* (《宋史 •食货志》), while cropland measurement data for six counties within the southern Jiangnandong Lu, including Xi, Xiuning, Qimen, Wuyuan, Jixi, and Yi, were extracted from the *Xinan Chronicles* (《新安志》). These measurements were the result of the projects *Land Measurement and Tax Equalization Law* and *Land Boundary Survey Law* that were carried out by Anshi Wang and Chunnian Li, respectively. And these measurement projects were completed in *Yuanfeng* (between AD 1072 and AD 1085) and *Chunxi Terms* (between AD 1174 and AD 1189), respectively.

S1.2 Household and population data

The household and population data estimates used in this study were derived from Wu and Ge [3]. These workers calibrated and estimated provincial household and population data for a number of periods during the Song, Liao, and Jin dynasties (i.e., between AD 980 and AD 989 and for AD 1078 during the Northern Song Dynasty, between AD 1162 and AD 1223 during the Southern Song Dynasty, and for AD 1078 and AD 1207 during the Liao and Jin dynasties, respectively) using a range of historical documents, including the *National Gazette* (《太平寰宇记》), *General Condition of Society and Natural Environment in Yuanfeng Term* (《元丰九域志》), *Monograph on Geography from the History of the Song Dynasty* (《宋史・地理志》), *Monograph on Military Camp Guarding from the History of the Liao Dynasty* (《辽史•兵卫志》), and *Monograph on Geography from the History of the Jin Dynasty* (《金史・地理志》).

Household numbers were converted to population data using conversion coefficients of 5.4 for the north and 5.2 for the south during the Song Dynasty, and of 6.5 for the Jin Dynasty, respectively. County level household data were supplemented by local chronicles from the Song Dynasty as well as the *Collected Texts of the Administrative Statues of the Song Dynasty* (《宋会要辑稿》).

S2 The Song Dynasty

This regime comprised the Northern and Southern Song dynasties. Of these, the first was established in AD 960 with its capital at Kaifeng Fu (a 'Fu' was a city unit during the Song Dynasty; Kaifeng Fu is the present-day city of Kaifeng and surrounding areas). Subsequent to AD 1126, the war between the Northern Song and Jin dynasties resulted in occupation of the north of the Huaihe River by the latter. And the Northern Song Dynasty moved its capital to Lin'an Fu (the present-day city of Hangzhou in Zhejiang Province) and established the Southern Song Dynasty in AD 1127.

S2.1 Accuracy assessment of cropland area data

Following the establishment of the Song Dynasty, a system of taxation and corvee was implemented by the government to increase national revenue and to consolidate its political power. The number of people in each household as well as their incomes and registered properties were used as taxable units in ancient China, and so were

afforded considerable attention by the local government [4]. Of these, the cropland area owned by each family formed the main basis of taxation during the Song Dynasty and so abundant historical records exist from this period. However, as land policy switched to allow free exchanges and mergers during the Song Dynasty [5], large areas of cropland were not registered to avoid agricultural taxes; thus, the recorded area of taxed-cropland at this time will be lower than actual area.

S3 The Liao Dynasty

The Liao Dynasty was created in northeastern China in AD 916 by the Khitan people, a minority who mainly subsisted by grazing, and had its capital in Linhuang Fu in Shangjing Lu (present-day Balinzuo Banner of the city of Chifeng, Inner Mongolia) (Fig. 1a). As the ruling class of this dynasty paid a great deal of attention to agricultural development, large numbers of people (including Han from the central plains and Bohai from the Liaodong region) moved into this region, especially to Shangjing Dao (a Liao Dynasty provincial level political unit) and Zhongjing Dao (the dynastic ruling center). The Liao Dynasty also controlled the Yanyun region subsequent to AD 986, creating Xijing and Nanjing Daos from an area that had previously belonged to the Northern Song Dynasty. These changes all led to a large agricultural population movement from the central plains into the Yanyun region that had finished by AD 1004 [6]. The region to the north of Yanshan Mountain then developed into a farming-pastoral zone that was self-sufficient throughout the period of the Liao Dynasty. The south of this region of China remained the dominant farming area throughout this period; its topographical environment and level of agricultural development were similar to Hedong and Hebei Lus during the period of the Northern Song Dynasty [7].

S3.1 Taxation systems

The Liao Dynasty government enforced two different tax regimes in the areas to the north (i.e., Shangjing, Zhongjing, and Dongjing Daos) and south (i.e., Nanjing and Xijing Daos) of Yanshan Mountain. In the north, taxes and corvee were levied on the basis of the number of Ding (i.e., men aged between 15 years old and 50 years old) in each household, while the tax system of the Northern Song Dynasty was followed in the south, with taxes and corvee collected based on household Ding and associated properties. These differences meant that the government paid more attention to

recording Ding numbers than cropland areas; indeed, Ding inventories were implemented on numerous occasions during this period [8] and were recorded for the mid-to-late Liao Dynasty (AD 1078) in *Monograph on Military Guarding from the History of the Liao Dynasty* (《辽史•营卫志》). Although the Ding of ordinary prefectural households were recorded for the five Fus (i.e., Linhuang, Dading, Liaoyang, Xijin, and Datong) and their surrounding areas during the Liao Dynasty, no historical records of cropland areas are known.

S3.2 Population compositions

Wu and Ge [3] analyzed Ding numbers in addition to other historical population records in order to estimate provincial agricultural and total nonagricultural populations during the Liao Dynasty (Fig. S1). And according to the findings of existing research [9] as well as historical records from the *Monograph on Camp Guarding from the History of the Liao Dynasty* (《辽史•营卫志》) show that the non-agricultural population at this time was mainly distributed within Shangjing and Zhongjing Daos. We therefore estimated provincial cropland in this study using population data.

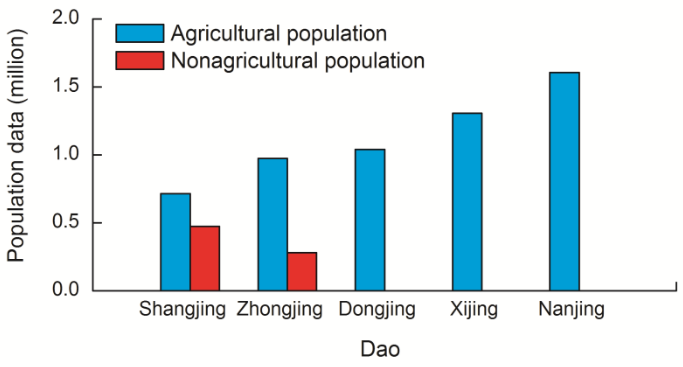

Figure S1. Provincial agricultural and nonagricultural population data for the mid-to-late Liao Dynasty.

S3.3 Human demands for cropland

To the north of Yanshan Mountain, farmers cultivated crops to support their basic life styles. Indeed, the presence of both agricultural and nonagricultural populations in this region led to both cultivation and grazing and a difference in demand for cropland area between the two. In the case of the agricultural population, the cropland demand for each person can be estimated using their grain demand for one year (i.e., 237.6 kg

for adults and 118.8 kg for minors) [10], crop yield per Song-mu (area unit of the Song Dynasty, 1 Song-mu=0.06 ha) (i.e., 29.7 kg in Shangjing and Zhongjing Daos, and 59.4 kg in Dongjing Dao) (Table S1), and cultivation mode (cropland at this time was cultivated every other year). Data show that human demands for cropland for each person were 12 Song-mu (about 0.7 ha) in Shangjing and Zhongjing Daos, and 6 Song-mu (about 0.35 ha) in Dongjing Dao. At the same time, the cropland demand of each person within the non-agricultural population was just one-tenth that of the agricultural population [7].

Data sources	Millet	Data sources	Millet
Beizhen County Annals	40–50	***Tieling County Annals***	70
Liaoyang County Annals	60–70	***Kaiyuan County Annals***	60–70
Liaozhong County Annals	60		

Table S1. Song-mu crop yield for selected states and counties of eastern Liaoning, China (unit: kg/ Song-mu). The tool used to measure grain during the Liao Dynasty was three times the size of that used during the Song Dynasty.

S4 The Jin Dynasty

The Jin Dynasty was founded in AD 1115 by the Jurchen people, a minority who subsisted by fishing and hunting in northeastern China. The capital of this dynasty was located in Huining Fu within Shangjing Lu, the present-day city of Harbin in Heilongjiang Province. This dynasty successively occupied the territories of the Liao Dynasty as well as the northern area of the Northern Song Dynasty, expanding its area of influence as far as the Huaihe River in the north by AD 1127 (Fig. 1b). During this period, large numbers of Han people from northern China migrated to the northeast to bolster the power of the Jin Dynasty; thus, after AD 1153, the capital was moved to Daxing Fu within Zhongdu Lu, and a segment of the Jurchen population migrated to northern China. As the population of the Jin Dynasty was consistently composed of a majority of Han and Jurchen nationalities, the government created two local administrative organizations to maintain power and manage the two groups, an ordinary prefectural household and Mengan Mouke. And two different taxation systems and land polices were also implemented to collect revenue and manage lands;

thus, we separately reconstructed Mengan Mouke and ordinary prefectural household cropland areas at the provincial level.

S4.1 Population compositions

In order to estimate separately provincial area under Mengan Mouke and ordinary prefectural households, we estimated the number of households subject to two systems. We initially extracted data on the number of provincial households subject to Mengan Mouke across the whole of this region in AD 1183 as well as for Shangjing Lu in AD 1193. These data were then combined with the population growth rate between AD 1183 and AD 1207 (7.1‰) [11], ratios of Mengan Mouke households in Shangjing, Dongjing, and Beijing Lus (6:3:1) [7], and the frequency distribution of these households across northern China [12] (Fig. S2). Previous work has shown that the number of such households can be converted to an overall population estimate by applying a conversion coefficient of 10 [3]. These historical records were used to calculate the number of Mengan Mouke households in some Lus in northern and northeastern China.

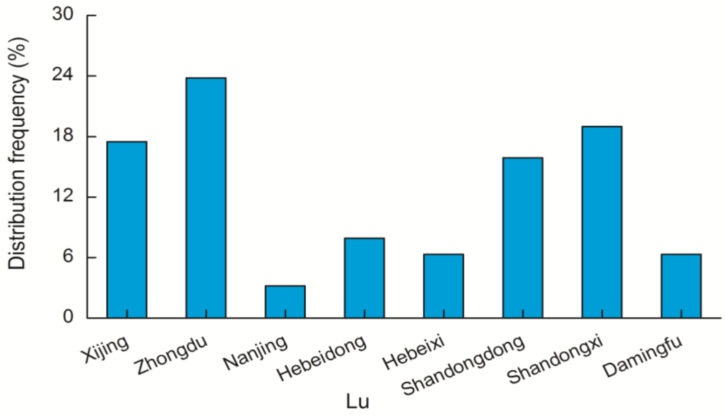

Figure S2. Distribution frequency of Mengan Mouke households in selected northern Chinese Lus.

The number of ordinary prefectural households was calculated by using the total number of households and Mengan Mouke households.

S4.2 Cropland allocation method for Mengan Mouke households

Although just provincial household numbers are present in historical documents from the Jin Dynasty, records relating to historical land use policies and the system of

taxation in Mengan Mouke also yielded important information. In Mengan Mouke, for example, cropland was allocated by the government, while the so-called ‘Cattle and Tool Tax’ stipulated that this should be allocated according to population level [13]. We know from the *Monograph on Food and Property from the History of the Jin Dynasty* (《金史·食货志》) that each group of 25 people was eligible for about 404 Song-mu (about 23.6 ha) under the Mengan Mouke system. We utilized these historical records to calculate the average per capita cropland area for each Mengan Mouke household at 16.16 Song-mu (about 0.94 ha).

S4.3 Per capita cropland area for ordinary prefectural households

Land occupation by ordinary prefectural households at this time was based on private ownership [13].Thus, the government collected agricultural as well as summer and autumn taxes (similar to the Song Dynasty) on the basis of the cropland area occupied by each ordinary household. This meant that local governments paid more attention to the registration and inventories of cropland area; during the *Shizong* (between AD 1161 and AD 1189) and *Zhangzong Terms* (between AD 1189 and AD 1208) periods, for example, the Jin Dynasty enacted a strict ‘General Survey and Evaluation’ system in order to survey property and expropriate tax. Between AD 1186 and AD 1187, the government performed this evaluation process three times [13], while due to the influence of wars and other factors, just a handful of cropland areas were recorded in AD 1189 in the *Monograph on Food and Property from the History of the Jin Dynasty* (《金史·食货志》). These data nevertheless encompassed some regions of Zhongdu and Hebei (present-day Jing-Jin and surrounding areas).

Based on cropland area and population data for this region, we initially analyzed the proportional relationship between the two variables to obtain a per capita cropland area of about 4.9 Song-mu (about 0.27 ha). We then analyzed the per capita cropland area for AD 1078 in order to obtain an estimate for the provincial cropland area of northern China; the results presented in Fig. S3 show that per capita values for cropland area across this region are distributed within a similar range of values. Thus, using the number of ordinary prefectural households as a proxy for per capita cropland area across northern China, we estimated corresponding provincial cropland areas. The number of provincial ordinary prefectural households was estimated using the number of provincial total households and Mengan Mouke households.

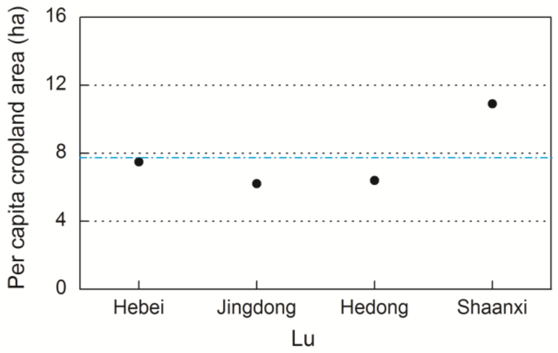

Figure S3. Calibrated per capita cropland area results for the northern region of the middle Northern Song Dynasty (AD1078); the horizontal blue dotted line denotes the average value of cropland area per household.

References

[1] Liang, F. Z. *The Statistics of Ancient Accounts, Land and Land Taxes of China: Preface*, 1-16 (in Chinese) (People's Publishing House, Shanghai, China, 1980).

[2] He, B. D. *The Textual Criticism and Evaluation of Chinese Ancient Land Records*, 1-114 (in Chinese) (China Social Sciences Press, Beijing, 1988).

[3] Wu, S. D. & Ge, J. X. *The History of Chinese Population*, Vol. 3, 1-665 (in Chinese) (Fudan University Press, Shanghai, China, 2000).

[4] Chen, M. G. & Zheng, X. M. The retrospect and prospect of Chinese taxes and labor taxes system history. *Hist. Res.* (01), 154-168 (in Chinese) (2001).

[5] Qi, X. *The Economic History of the Song Dynasty*, Vol. 1 (in Chinese) (Shanghai People's Publishing House, Shanghai, China, 1987).

[6] Wu, S. D. & Ge, J. X. *The History of Immigration in China*, Vol. 4 (in Chinese) (Fujian People`s Publishing House, Fuzhou, China, 1997).

[7] Han, M. L. *Agricultural Geography of the Liao and Jin Dynasty* (in Chinese) (Social Sciences Academic Press, Beijing, China, 1999).

[8] Han, G. H. & Zhang, Q. H. A study of the type of household in the Liao Dynasty. *Northern Cultural Relics* (03), 71-73 (in Chinese) (2003).

[9] Wu, Y. H. An investigation of the Woluduo, an organization of the Liao Dynasty. *Hist. Res.* (02), 51-62 (in Chinese) (2000) .

[10] Cheng, M. S. Living standard and monetary value of Song Dynasty. *J. Hist. Sci.* (03), 100-111 (in Chinese) (2008).

[11] Liu, P. J. A study of the registered resident in the Jin Dynasty. *J. Chinese Hist. Res.* (02), 86-96 (in Chinese) (1994).

[12] Tsugio, M. *A Study of the Jurchen in the Jin Dynasty*, 160-161 (in Chinese) (Heilongjiang People's Publishing House, Harbin, China, 1984).

[13] Qi, X. & Qiao, Y. M. *The History of Economy in the Liao, Xixia, and Jin Dynasty* (in Chinese) (Hebei University Press, Baoding, China, 1994).
